# An Alternate Approach to Generate Induced Pluripotent Stem Cells with Precise CRISPR/Cas9 Tool

**DOI:** 10.1155/2022/4537335

**Published:** 2022-09-22

**Authors:** Nasir Javaid, Sangdun Choi

**Affiliations:** ^1^Department of Molecular Science and Technology, Ajou University, Suwon 16499, Republic of Korea; ^2^S&K Therapeutics, Ajou University Campus Plaza 418, 199 Worldcup-ro, Yeongtong-gu, Suwon 16502, Republic of Korea

## Abstract

The induced pluripotent stem cells (iPSCs) are considered powerful tools in pharmacology, biomedicine, toxicology, and cell therapy. Multiple approaches have been used to generate iPSCs with the expression of reprogramming factors. Here, we generated iPSCs by integrating the reprogramming cassette into a genomic safe harbor, CASH-1, with the use of a precise genome editing tool, CRISPR/Cas9. The integration of cassette at CASH-1 into target cells did not alter the pattern of proliferation and interleukin-6 secretion as a response to ligands of multiple signaling pathways involving tumor necrosis factor-*α* receptor, interleukin-1 receptor, and toll-like receptors. Moreover, doxycycline-inducible expression of *OCT4*, *SOX2*, and *KLF4* reprogrammed engineered human dermal fibroblasts and human embryonic kidney cell line into iPSCs. The generated iPSCs showed their potential to make embryoid bodies and differentiate into the derivatives of all three germ layers. Collectively, our data emphasize the exploitation of CASH-1 by CRISPR/Cas9 tool for therapeutic and biotechnological applications including but not limited to reprogramming of engineered cells into iPSCs.

## 1. Introduction

Induced pluripotent stem cells (iPSCs) are being used in regenerative medicine, disease modeling, drug screening, and cell-based therapies [1–3]. Extensive demand of iPSCs provoked to invent as well as revolutionize the existing approaches in order to enhance their efficient generation. The expression of reprogramming factors from the integrated transgene reprograms specialized cells into iPSCs. However, reported viral and nonviral integrative strategies are not considered safer [4, 5]. It can be overcome by using a more precise and efficient genome editing tool, i.e., CRISPR/Cas9 [6, 7]. The genome editing efficiency can also be influenced by the genomic context of target site [8, 9] and features of the donor cassette [10, 11]. Hence, it is also necessary to estimate the efficiency of insertion-deletion mutations (indels) as well as knock-in at a particular genomic safe harbor (GSH). The CRISPR/dCas9 activator systems had been already used to generate iPSCs via activation of the transcriptionally silenced endogenous reprogramming genes, i.e., *OCT4*, *SOX2*, *KLF4*, *c-MYC*, and *LIN28* [12–14]. The Yonglun lab used a lentiviral transduction system to generate iPSCs by integrating dCas9-VP64 and MS2-P65-HSF1 along with a polycistronic cassette expressing *OCT4*, *KLF4*, *SOX2*, and *c-MYC* into normal human dermal fibroblasts (HDFs) [15]. However, the paired double-nicking CRISPR/Cas9 system has not been used yet to integrate reprogramming cassette and generate iPSCs from human cells. The catalytic inactivation of one of the nuclease domains in nickase Cas9 makes it more specific in cutting the target site of genome [16, 17].

The transgenes inserted at random site can reciprocally interact with the host genome which can lead to complete silencing or attenuated expression of the transgene [18, 19]. More critically, it can also affect the expression of endogenous genes positioned in neighborhood or at a distance via long-range interactions. Any dysregulation in key endogenous genes can dramatically affect the cellular behaviors [20]. To overcome these deleterious effects and to ensure accurate expression of transgene, a suitable GSH is necessary [21]. Among GSHs, adeno-associated virus site 1 (AAVS1) [22, 23], chemokine (C-C motif) receptor 5 (CCR5) [24], and ROSA26 [25] have been extensively exploited in human cells, while each one of them shows distinct expression patterns of integrated transgene based upon the genomic context [26, 27]. Moreover, none of these GSHs completely fulfill the criteria to be a GSH such as (i) 50 kb distance from any gene, (ii) 300 kb distance from cancer-related gene and microRNA (miRNA), and (iii) location outside a transcription unit and ultraconserved regions [27]. Previously, an alternate GSH (chromosome 1: position 188,083,272; named as CRISPR/Cas9-accessible safe harbor-1 (CASH-1) in our study) is found by targeting bone marrow mesenchymal stem cells (MSCs) and skin fibroblasts with the nonspecific lentiviral system. The expression of integrated reprogramming factors generated iPSCs from both target cells; however, the clones with the accurate integration at CASH-1 were only from MSCs [28]. Hence, it is not clear from their study whether clones generated from the skin fibroblasts were because of the sole expression of reprogramming factors or due to the additional perturbation in the expression of other genes. This might underestimate the therapeutic usage of CASH-1 as GSH, so there is a need to test it further.

Eukaryotic cells pass through multiple stages in order to complete a cell cycle where each stage is controlled by the particular cyclins. The expression of these cyclins (such as cyclin D1) is dependent on the activity of multiple transcription factors such as NF-*κ*B and AP-1 [29, 30]. These transcription factors are activated with the recognition of a particular ligand by its associated receptor such as tumor necrosis factor-*α* (TNF-*α*) receptor, interleukin-1 (IL-1) receptor, and toll-like receptors (TLRs) [31–34]. Any dysregulated activation of these factors affects the expression of cyclins which can affect the proliferation rate of cells [35]. Besides this, proliferation can also be affected by the integration of transgene at an unsafe loci [36, 37] as well as AAVS1 [38, 39]. Thus, it is necessary to evaluate the proliferative behavior of cells after integrating transgene into CASH-1 GSH.

In the present study, we optimized the precise integration of a long, reprogramming transgene-cassette into the CASH-1 locus by using a paired nicking CRISPR/Cas9 system. We demonstrated that integration at CASH-1 does not affect the ability of transgenic cell lines to proliferate as well as respond to ligands of various cellular signaling pathways. We also showed that controlled expression of *OCT4*, *SOX2*, and *KLF4* can reprogram fibroblasts and HEK293T cells into iPSCs.

## 2. Materials and Methods

### 2.1. Design and Cloning of gRNAs

Six target sites were selected within CASH-1 region adjacent to 5′-NGG-3′ PAM sequence, which is recognized by *Streptococcus pyogenes*–derived Cas9 (SpCas9) (Supplementary Figure 1) [40]. The Cas-OFFinder tool did not predict any off-target within the selected CASH-1 region, i.e., chr1: 188,082,217–188,083,803 (Supplementary Table 1). We designed six 20-nucleotide-long gRNAs (g1-g6) specific to each target site (Supplementary Table 2). Vector pX330-U6-Chimeric_BB-CBh-hSpCas9 (Addgene #42230) was digested with BbsI-HF (NEB #R3539) and treated with calf intestinal alkaline phosphatase (NEB #M0290) according to the manufacturer's protocol. At the 10 *μ*M final concentration, oligonucleotides of each designed gRNA were annealed in a mixture of ATP (1× final concentration; NEB), T4 PNK buffer (1× final concentration; NEB), and T4 PNK (polynucleotide kinase, 10 U; NEB). The annealing conditions on a thermocycler were adjusted as follows: 95°C for 5 min followed by a -5°C/(3 min) ramp down to 65°C; 63°C for 3 min followed by a -3°C/(3 min) ramp down to 27°C, and a final incubation at 25°C for 10 min. The PNK-treated annealed oligonucleotides were diluted to a final concentration of 1 *μ*M and ligated into the above-mentioned digested vectors by means of the T4 DNA ligase enzyme (NEB #M0202) at 16°C for 16 h. DH10B (Invitrogen) competent cells were transformed with constructs followed by spreading on ampicillin-containing (100 *μ*g/ml) 2XYT agar plates, and grown overnight at 37°C. One to two colonies were randomly picked for colony PCR analysis (Supplementary Figure 2(a)) with a forward primer specific to the U6 promoter sequence (Supplementary Table 3) and a reverse primer specific to the relevant cloned gRNA (Supplementary Table 2). The plasmid DNA was isolated from the clones (1B, 2B, 3A-6A) using the PureLink HiPure Plasmid DNA Purification Kit (Invitrogen) according to the manufacturer's instructions and was processed for confirmation by Sanger sequencing (Macrogen, Korea; Supplementary Figure 2(b)).

Similarly, pX335-U6-Chimeric_BB-CBh-hSpCas9n(D10A) (Addgene #42335) plasmid was digested and ligated to annealed g1 and g2 oligonucleotides followed by ampicillin selection. Four colonies were randomly picked for colony PCR analysis (Supplementary Figures 2(c)), and two of them (1An and 2An) were further confirmed by Sanger sequencing (Supplementary Figure 2(d)). These g1 and g2 containing constructs are deposited at Addgene as pX335-U6-Chimeric_BB-CBh-hSpCas9n(D10A)_g1_CASH-1 (Addgene # 188975) and pX335-U6-Chimeric_BB-CBh-hSpCas9n(D10A)_g2_CASH-1 (Addgene # 188976), respectively.

### 2.2. Cell Culture and Reagents

The HDFs (ATCC) and HEK293T (ATCC) cells were maintained in DMEM (Gibco) supplemented with 10% of FBS (Gibco), 1% of a penicillin/streptomycin solution (Gibco), and 100 *μ*g/ml Normocin (InvivoGen). The cells were grown in a humidified atmosphere with 5% of CO_2_ at 37°C and passaged three times a week. For cell cycle synchronization, cells at 70% confluency were kept in a serum-free medium for 48–72 h before using them.

### 2.3. Off-Target Prediction

The off-targets of all gRNAs were predicted by using the Cas-OFFinder tool [41]. The off-targets were found for *Streptococcus pyogenes* Cas9 enzyme (5′-NGG-3′ protospacer adjacent motif sequence) with 3 mismatches in GRCh38/hg38 target genome of *Homo sapiens*. The complete list of off-targets is mentioned in supplementary table 1.

### 2.4. The T7E1 Endonuclease Assay

HEK293T cells were seeded at 10^5^ cells per well in a 12-well plate and reverse-transfected with a 30 min preincubated mixture of 12 *μ*l of Lipofectamine 2000 (Invitrogen) and 6 *μ*g of either a respective gRNA plasmid (gRNA 1–6) or a control plasmid (plasmid not encoding gRNA), separately. After 10 h of incubation, the medium was replaced with a fresh complete medium, and the transfected cells were further incubated for 72 h. The genomic DNA from the cells of each treated well was separately isolated with the DNA Tissue Kit (Kurabo, Japan) according to the manufacturer's protocol. The genomic DNA flanking the gRNA target site was PCR amplified with the first set of T7E1 primers. The PCR product was gel-purified using the PureLink™ Quick Gel Extraction Kit (Invitrogen) according to the manufacturer's protocol. The purified product was then PCR amplified with the second set of T7E1 primers followed by gel-purification as described above. The primer sequences are listed in supplementary table 3. A purified PCR product (1 *μ*g) was mixed with 3.5 *μ*l of 10× NEB 2 buffer (NEB), and double-distilled water was added to a final volume of 35 *μ*l for the formation of heteroduplexes via the reannealing process on the thermocycler (Applied Biosystems): 95°C for 15 min, 25°C for 1 min, 95°C for 10 min, 95 to 87°C ramping at -2°C/5 s, 85°C for 30 s, 85 to 77°C ramping at -2°C/5 s, 75°C for 30 s, 75 to 67°C ramping at -2°C/5 s, 65°C for 30 s, 65 to 57°C ramping at -2°C/5 s, 55°C for 30 s, 55 to 47°C ramping at -2°C/5 s, 45°C for 30 s, 45 to 37°C ramping at -2°C/5 s, 35°C for 30 s, 35 to 27°C ramping at -2°C/5 s, and 25°C for 30 s. After the reannealing process, the products were treated with 4.5 U of T7 endonuclease I (NEB #M0302) for 20 min at 37°C and analyzed on a 2% ethidium bromide (Invitrogen)-stained agarose gel. The ImageJ software (version 1.47 V) was utilized to quantify the band intensities. The indel percentage for each target site was determined via the formula 100 × (1 − (1 − (*b* + *c*)/(*a* + *b* + *c*))1/2), where *a* is the intensity of the undigested PCR product and *b* and *c* are intensities of each cleaved band.

### 2.5. Reprogramming-Plasmid Construction

For the integrated expression of reprogramming factors, the donor cassette was designed to be specific to the CASH-1 site. Briefly, open reading frames (ORFs) of *OCT4*, *SOX2*, *KLF4*, and *EGFP* were used to generate a single polycistronic expression cassette for separate expression of each of these genes. This was done by removing the stop codons at the end of each gene (except *EGFP*) and by separating them with the insertion of 2A peptide sequences for ribosome skipping. *EGFP* was included to serve as an expression marker. For controlled expression of the reprogramming factors, the promoter sequence of the Tet-on system was used. The blasticidin (*BSD*) resistance gene was inserted as a eukaryotic selection marker. For precise integration at CASH-1, the donor cassette was flanked by 800 bp left- and right-hand sequences homologous to CASH-1. The above-mentioned fragments were also separated by various restriction sites, separators, and tails. The whole designed cassette was inserted into pUC57-Amp serving as a cloning vector. The customized gene synthesis and cloning service of Synbio Technologies (Monmouth junction, NJ, USA) were hired to construct the donor plasmid. This reprogramming donor plasmid is deposited at Addgene as pTetO-hOSK-CASH-1 (Addgene # 188977).

### 2.6. Donor Cassette Knock-in Optimization

Unsynchronized and cell cycle–synchronized HEK293T cells were transfected at a 5 : 1, 1 : 1, or 1 : 3 molar ratio of the gRNA/Cas9 plasmid to the donor plasmid. Both circular and linear forms of the donor plasmid were tested with the wild-type and nickase versions of Cas9, individually. To linearize the donor plasmid, it was double-digested with BamHI-HF (NEB #R3136) and EcoRV-HF (NEB #R3195) and gel-purified via the PureLink™ Quick Gel Extraction Kit (Invitrogen) according to the manufacturer's protocol. For transfection, the cells were grown to 70% confluency in a 12-well plate, and then a 30-min-preincubated mixture of 3 *μ*l of Lipofectamine 2000 (Invitrogen) and 1.5 *μ*g of total DNA in Opti-MEM (Gibco) was applied dropwise to the cells. After 6 h of treatment, the medium was replaced with a fresh complete DMEM medium, and the transfected cells were further grown for 72 h. Transfection-positive cells were selected in a medium supplemented with 10 *μ*g/ml blasticidin (InvivoGen) for 2 weeks.

Unsynchronized and cell cycle–synchronized HDFs were transfected with the gRNA/Cas9 plasmid and the donor plasmid at a 1 : 1 or 1 : 3 molar ratio. Both circular and linear forms of the donor plasmid were tested with the nickase version of Cas9, separately. The procedures for linearization, transfection, and selection were similar to those for HEK293T cells. The genetically modified HEK293T and HDF stable cell lines were named as HEK293T-OSK and HDF-OSK, respectively.

### 2.7. Junction PCR

Genomic DNA was isolated from the selected cells with the help of the DNA Tissue Kit (Kurabo, Japan) according to the manufacturer's protocol. The 5′ and 3′ junction PCRs were carried out with the GoTaq Green Master Mix (Promega #M7122) and relevant primers (Supplementary Table 3). The thermocycler program was set as follows: 1 cycle at 95°C for 5 min; 30 cycles at 95°C for 30 s, 58°C for 30 s, and 72°C for 3 min; 1 cycle at 72°C for 10 min; and holding at 4°C. The amplicons were analyzed by electrophoresis on a 1% ethidium bromide (Invitrogen)-containing agarose gel and visualized under UV light.

### 2.8. Flow Cytometry

To enrich GFP-expressing cells, stable cell lines were grown to 95% confluency in a culture medium supplemented with 2 *μ*g/ml DOX (Sigma) and 10 *μ*g/ml blasticidin (InvivoGen). On the day of sorting, the cells were trypsinized with a 0.05% trypsin-EDTA solution (Gibco) and centrifuged at 415 rcf for 3 min, and their concentration was adjusted to 10^7^ cells/ml after cell counting. The cells were sorted based upon the intensity of a GFP signal by flow cytometry. The sorted cells were then collected into a new tube containing a medium, centrifuged at 415 rcf for 3 min, and seeded in a 6 cm dish containing a growth medium.

To analyze cell-surface pluripotency markers, iPSC clones derived from HDF-OSK and HEK293T-OSK cells were resuspended (2 × 10^6^ cells/ml) in ice-cold PBS, 1% sodium azide, and 10% FBS. The cell suspension (250 *μ*l) was added into each tube; fixed with paraformaldehyde (4%) for 15 min on ice; and treated with anti-SSEA4 (Abcam) and anti-TRA1-60 (Abcam) primary antibodies (2 *μ*g/ml in 3% BSA/PBS) for 1 h at room temperature. Cells were washed three times with ice-cold PBS and treated with Alexa Fluor 488– or Alexa Fluor 647–conjugated secondary antibodies (5 *μ*g/ml in 3% BSA/PBS; Invitrogen) for 30 min at room temperature. Cells were washed three times with PBS; resuspended in ice-cold PBS, 1% sodium azide, and 3% BSA; and stored until analysis. Mouse IgG3 monoclonal antibody (Abcam) served as an isotype control. Flow cytometry analysis was done in FACSAria III, BD Biosciences instrument, and data processing was performed in the FACSDiva software (BD Biosciences).

### 2.9. Cell Viability Assay

The cell viability was determined with (3-(4,5-dimethylthiazol-2-yl)-2,5-diphenyltetrazolium bromide (MTT) assay (Sigma-Aldrich) as mentioned before [42]. Briefly, HEK293T-OSK and HEK293T cells were seeded in 96-well plate at the density of 1 × 10^4^ cells/well. Similarly, HDF-OSK and HDF were seeded at the density of 0.5 × 10^4^ cells/well. After growing them overnight, cells were left untreated (control); treated with immunostimulatory ligands; and/or DOX followed by further incubating them for overnight in humidified incubator. Next day, the medium was replaced with complete medium containing 10% MTT solution (100 *μ*l/well), and cells were incubated for 3 h. The formazan crystals were dissolved by replacing the solution with DMSO (100 *μ*l/well; Sigma-Aldrich Corp., St. Louis, MO, USA) and incubating for further 30 min. Finally, absorbance was measured at 540 nm on a Synergy™ HTX multimode microplate reader (BioTek Instruments, Winooski, VT, USA). The used immunostimulatory ligands included rhTNF-*α* (1 ng/ml; Miltenyi Biotec, Auburn, CA, USA), rhIL-1*β* (50 ng/ml; R&D, Minneapolis, MN), lipopolysaccharide (LPS; 100 ng/ml; InvivoGen, San Diego, CA), and polyinosinic: polycytidylic acid (pIC; 1 *μ*g/ml; InvivoGen, San Diego, CA).

### 2.10. Cytokine Detection Assay

The level of secreted cytokines was determined by using the enzyme-linked immunosorbent assay (ELISA). Briefly, serum was collected from the respective cells after the completion of above-mentioned treatment and processed for the detection of interleukin-(IL-)6 with IL-6 human uncoated ELISA kit (Thermo Fisher Scientific, Inc.) by following the manufacturer's instructions. The absorbance in the plate was read on a Synergy™ HTX multimode microplate reader (BioTek Instruments, Winooski, VT, USA).

### 2.11. Generation and Maintenance of iPSCs

HDF-OSK and HEK293T-OSK cells were seeded in a gelatin-coated (0.2%) 6-well plate at a density of 9 × 10^4^/well and grown in a normal medium (as described above) for 24 h. Next day, medium was supplemented with DOX (2 *μ*g/ml) and blasticidin (10 *μ*g/ml). After 24 h, the medium was replaced with a reprogramming medium: Knockout DMEM/F12 (Gibco) supplemented with 20% of knockout serum replacement (KSR; Gibco), 2 mM l-glutamine (Gibco), 0.1 mM nonessential amino acids (Gibco), 0.1 mM *β*-mercaptoethanol (Gibco), 50 U/ml penicillin and 50 *μ*g/ml streptomycin (Hyclone), 10 ng/ml basic fibroblast growth factor (bFGF; PeproTech), 2 *μ*g/ml DOX, and 10 *μ*g/ml blasticidin. The medium was refreshed daily until the emergence of morphological changes of the cells. The morphologically changed colonies were manually picked and transferred to a Matrigel-coated 6-well plate containing the TESR-E8 maintenance medium (Stemcell) and 10 *μ*M ROCK inhibitor (Stemcell). The next day, the medium was replaced with a fresh maintenance medium without the ROCK inhibitor, and the latter medium was refreshed every 2 days until the colonies grew enough to be subcultured. For subculturing, a mechanical approach with a cell scraper was used.

### 2.12. *In Vitro* Differentiation of iPSCs

We employed the hanging drop method for the formation of embryoid bodies. Briefly, harvested iPSCs were counted, their concentration was adjusted to 1000 cells per 20 *μ*l of the culture medium without bFGF, and the cells were incubated for 2 days in a hanging drop on the lid of a petri dish. The generated embryoid bodies were suspension-cultured for 5 days in DMEM supplemented with 10% of FBS, 1 mM L-glutamine, and 1% of a solution of nonessential amino acids. The embryoid bodies were then transferred to 12-well plates and grown on gelatin-coated cover slips for another 7 days with refreshment of the medium every other day. Finally, the cover slips were removed and processed for immunocytochemical analysis of germ layers.

### 2.13. Immunocytochemistry

The iPSCs or embryoid bodies that spontaneously differentiated were grown in a suitable medium on glass coverslips placed in a 12-well plate. The grown cells were washed with 1× PBS; fixed and permeabilized in chilled methanol (Samchun Chemicals, Korea) for 10 min; washed with PBS; and blocked with a 3% BSA solution in PBS (Thermo Fisher Scientific, Inc.) for 30 min. After that, the cells were incubated with a primary antibody at 4°C overnight. The next day, the cells were rigorously washed and incubated with Alexa Fluor 488– or Alexa Fluor 546–conjugated secondary antibodies (Invitrogen, Carlsbad, CA, USA) for 1 h at room temperature. After a wash with PBS, nuclei were stained with a Hoechst 33258 solution (5 *μ*M; Sigma-Aldrich) for 10 min. For staining of pluripotency markers of iPSCs, the following primary antibodies were used: anti-TRA1-60 (1 : 500; Abcam), anti-SSEA4 (1 : 500; Abcam), anti-OCT4A (1 : 1000; Cell Signaling Technology), and anti-NANOG (1 : 1000; Cell Signaling Technology). To detect germ layer markers of differentiation, the following primary antibodies were used: anti-AFP (for endoderm; 1 : 100; Santa Cruz Biotechnology), anti-SMA (for mesoderm; 1 : 250, Sigma), and anti-TUJ-1 (for ectoderm; 1 : 250; Abcam). All images were captured using the fluorescence microscope (Olympus IX53; Olympus Corporation, Tokyo, Japan).

### 2.14. RT-PCR and Quantitative RT-PCR

Total RNA was isolated using the TRI Reagent® Solution (Sigma-Aldrich) according to the manufacturer's protocol. Impurities consisting of genomic DNA were removed by processing the RNA samples with the TURBO DNA-free™ Kit (Thermo Fisher Scientific Inc.). The purified RNA was reverse-transcribed using the iScript cDNA Synthesis Kit (Bio-Rad). For RT-PCR, the GoTaq® Green Master Mix (Promega) was employed to amplify target genes under the following thermal cycling conditions: 95°C for 5 min followed by 25 cycles of 95°C for 10 s, 58°C for 10 s, and 72°C for 20 s. For quantitative RT-PCR, the Light Cycler 480 SYBR Green I Master Mix (Roche) was used to quantify the expression of target genes as mentioned before [43]. The used thermal cycling conditions were 95°C for 5 min, followed by 45 cycles of 95°C for 10 s, 58°C for 10 s, and 72°C for 20 s. The amount of each mRNA was normalized to that of *GAPDH* mRNA, and the relevant mRNA of untreated cells served as a negative control. Indicated error bars are the averages with standard deviations of three independent experiments. The primers used are listed in supplementary table 3.

### 2.15. Western Blotting

The cells were harvested by centrifugation, and total protein was extracted with the Whole-Protein Extraction Solution (M-PER; Thermo Fisher Scientific Inc.) supplemented with a protease and phosphatase inhibitor cocktail (Thermo Fisher Scientific Inc.). The mixture was centrifuged at 16,000 × *g* for 10 min, and the protein in the supernatant was subjected to quantification with the BCA Kit (Sigma-Aldrich Co. LLC). Protein samples (20 *μ*g) were loaded onto an SDS-polyacrylamide gel; transferred to a Hybond ECL nitrocellulose membrane (Amersham Pharmacia Biotech, Inc., Piscataway, NJ, USA); blocked with 5% nonfat dried milk; and immunoblotted at 4°C overnight with primary antibodies against OCT4A (2890, Cell Signaling Technology), SOX2 (ab97959, Abcam), KLF4 (ABS1514, Merck), and *α*-tubulin (AbC-2001, AbClon). The next day, membranes were washed with PBST; incubated with horseradish peroxidase–conjugated anti-mouse or anti-rabbit secondary antibodies (Thermo Fisher Scientific Inc.); treated with a SuperSignal West Pico ECL solution (Thermo Fisher Scientific Inc.); and visualized on a Fuji LAS-3000 system (Fujifilm, Tokyo, Japan). The *α*-tubulin served as a loading control.

### 2.16. Statistical Analysis

The statistical analysis was performed by using PRISM (GraphPad Software, San Diego, CA, USA) statistical software. Multiway analysis of variance (ANOVA) with Tukey's test was performed to compare the viability and immunological responses of wild-type (HEK293T and HDF) and genetically modified stable cell lines (HEK293T-OSK and HDF-OSK) in the presence or absence of DOX as well as immunostimulatory ligands. The *P* value <0.05 was considered statistically significant.

## 3. Results

### 3.1. Validation of Designed Guide-RNAs

**Figure 1:**
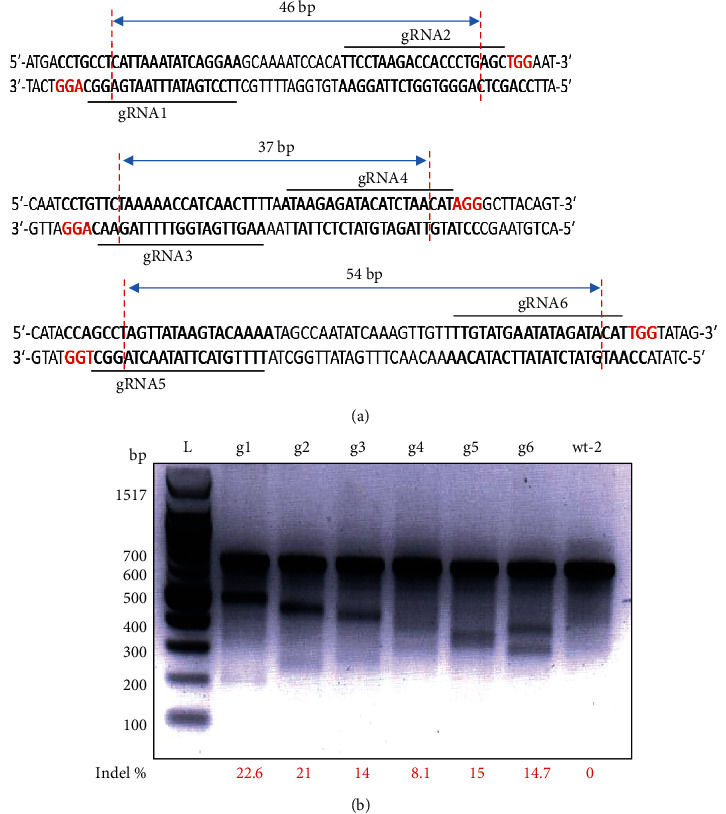
Validation of CASH-1–specific gRNAs. (a) Schematic representation of designed gRNAs and their target. (b) The T7E1 endonuclease assay after transfection of a relevant gRNA into HEK293T cells. The ImageJ software was employed for the calculation of indel percentages. L: 100-bp ladder.

To validate genome editing of CASH-1, six constructs with constitutive expression of a specific gRNA (g1-g6; Figure 1(a)) as well as human codon-optimized SpCas9 enzyme were designed and transfected separately into HEK293T cells. After 72 h of transfection, genomic DNA was isolated and processed for T7 endonuclease I assay in order to calculate indel percentage for each gRNA. We calculated values of indel percentages as 22.6%, 21%, 14%, 8.1%, 15%, and 14.7% for g1, g2, g3, g4, g5, and g6, respectively (Figure 1(b)). These values indicate the ability of a given gRNA to edit its target site [44]. However, the calculated values of indel percentage might not be the exact genome editing efficiency of these gRNAs as T7E1 assay has its own detection limitations [45].

### 3.2. Knock-in of Reprogramming-Cassette

**Figure 2:**
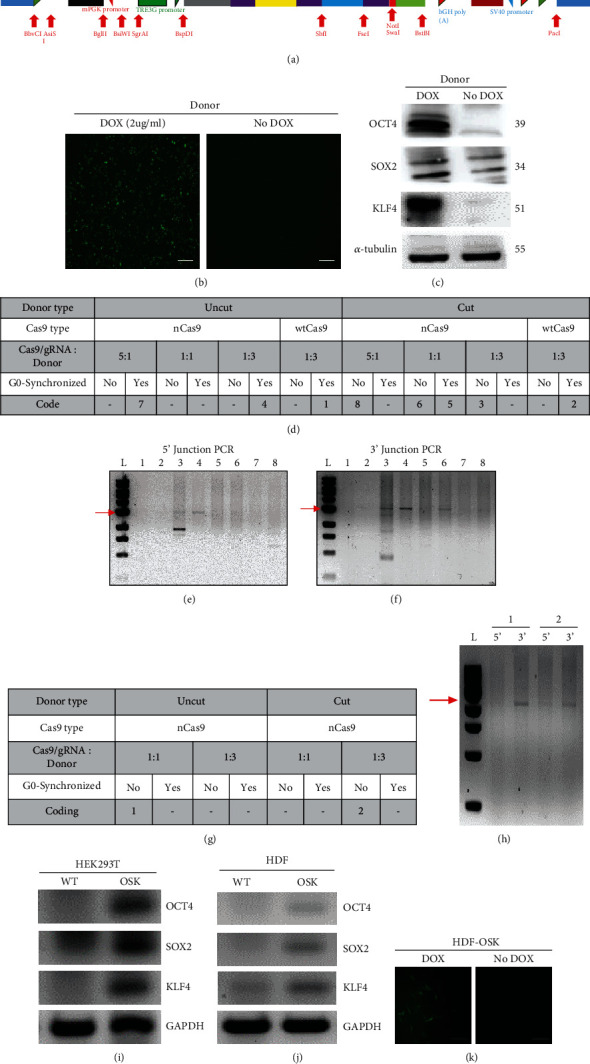
Knock-in and validation of reprogramming donor cassette. (a) Design of the DOX-inducible polycistronic expression cassette of reprogramming factors *OCT4*, *SOX2*, and *KLF4*. Each component is named and labeled with a different color. The red arrows under the cassette represent the restriction sites, and the cassette is flanked by sequences homologous to CASH-1. The DNA sequence of the whole cassette is shown in supplementary figure 3. (b) Detection of a GFP signal in donor-transfected HEK293T cells with or without DOX induction. Scale bar: 500 *μ*m. (c) Confirmation of protein expression of the reprogramming factors in the donor-transfected HEK293T cells with or without DOX induction. (d) Tabular plan followed for optimization of the donor cassette knock-in in HEK293T cells. (e and f) The 5′ (e) and 3′ (f) junction PCR assays of the template genomic DNA isolated from selected HEK293T colonies. (g) Tabular plan followed for optimizing the donor cassette knock-in in HDFs. (h) 5′ and 3′ junction PCR assays of the template genomic DNA isolated from selected HDF colonies. (i and j) Confirmation of mRNA expression of each reprogramming factor in HEK293T-OSK (i) and HDF-OSK cells (j) in comparison with respective normal cells (WT), where GAPDH mRNA served as a loading control. (k) Confirmation of GFP expression in HDF-OSK cells with or without DOX induction for 24 h under fluorescence microscope. Scale bar: 200 *μ*m. L: 1-kbp ladder.

To generate iPSCs, we constructed a reprogramming cassette (donor) in which expression of reprogramming factors (OCT4, SOX2, and KLF4) and expression marker (green fluorescent protein (GFP)) is controlled by a third-generation doxycycline-responsive element (TRE3G; Figure 2(a) and Supplementary Figure 3). The expression of reverse-Tet repressor protein (rTetR) is driven by a strong constitutive promoter (mPGK) which is oriented in opposite direction to minimize leaky expression of reprogramming factors without doxycycline. The rTetR protein can only bind to TRE3G site in the presence of tetracycline or its derivatives such as DOX. This binding leads to the expression of downstream genes [46]. Moreover, DNA sequences of each reprogramming factor and expression marker in our cassette were separated from each other by 2A sequences of virus origin in order to get their separate expression from the same promoter via ribosome skipping [47, 48]. For precise integration at CASH-1, reprogramming cassette is provided with sequences homologous to the CASH-1 locus. To select a concentration of DOX for induced expression of donor, HEK293T cells were transfected with donor plasmid (1.5 *μ*g) and treated with various concentrations of DOX (1-5 *μ*g/ml) for 48 h. It was followed by detecting the expression of GFP marker under fluorescence microscope. We detected stronger signal of GFP at concentrations of DOX higher than 1 *μ*g/ml which remained almost consistent from 2 *μ*g/ml to 5 *μ*g/ml concentration (Supplementary Figure 4 and Figure 2(b)). It led us to choose 2 *μ*g/ml concentration of DOX for next experiments. Moreover, GFP signal was only observed in the presence of DOX. Similarly, we observed separate expression of OCT4, SOX2, and KLF4 by western blotting only in the presence of DOX (Figure 2(c)). These results indicate individual expression of each protein from a single promoter in DOX-inducible manner.

**Figure 3:**
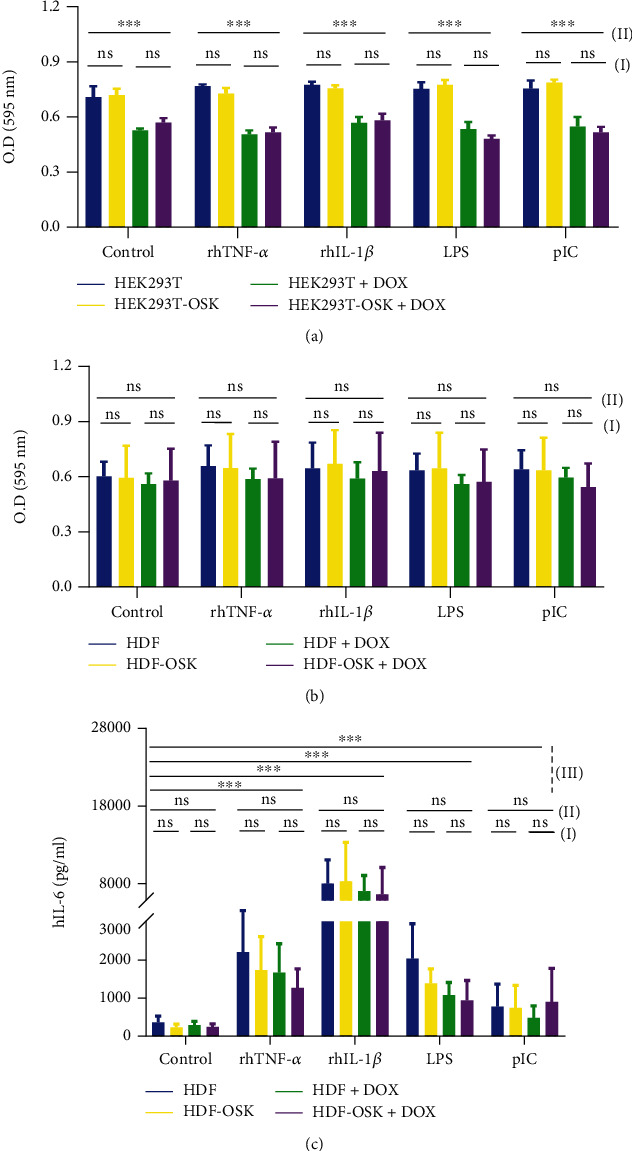
Proliferative and immunological validation. (a, b) Cell viability of HEK293T-OSK and HEK293T (a) as well as HDF-OSK and HDF (b) were measured by MTT assay. (c) Supernatant was collected from HDF-OSK and HDF cells and analyzed for the amount of secreted interleukin-(IL-)6 with respective ELISA kit. The absorbance was measured by Synergy™ HTX multimode microplate reader. The statistical comparison among variables was done by multiway analysis of variance (ANOVA) with Tukey's test in PRISM (GraphPad Software, San Diego, CA, USA) statistical software. The *P* values <0.05 (∗), <0.01 (∗∗), and <0.001 (∗∗∗) were considered statistically significant. The used immunostimulatory ligands included rhTNF-*α* (1 ng/ml), rhIL-1*β* (50 ng/ml), lipopolysaccharide (LPS; 100 ng/ml), and polyinosinic: polycytidylic acid (pIC; 1 *μ*g/ml). ns = nonsignificant.

To integrate reprogramming cassette at CASH-1, we selected paired double-nicking CRISPR/Cas9 system with nicked Cas9 (nCas9) and two gRNAs (g1 and g2) in order to enhance specific integration [49]. Knock-in of reprogramming cassette was optimized for HEK293T cells (Figure 2(d)) and HDFs (Figure 2(g)) by considering the factors affecting knock-in efficiency of a transgene such as the conformation of the donor [50], the ratio of the Cas9/gRNA plasmid to the donor plasmid [51], and a cell cycle phase of the target cell [52]. We also included a single gRNA (g1) with wild-type Cas9 (wtCas9) for HEK293T cells. Collectively, HEK293T cells were first cotransfected with Cas9/gRNA and donor plasmids in 16 different combinations (Figure 2(d)). After 72 h of transfection, HEK293T cells were selected in a blasticidin-containing medium for 2 weeks. Out of 16, 8 combinations (50%) allowed transfected HEK293T cells to survive under blasticidin selection pressure. These combinations were assigned a numerical code (1-8), while others with dead cells were given a minus (-) sign (Figure 2(d)). Next, blasticidin-resistant HEK293T cells were screened by junction PCR for the targeted integration of donor cassette at CASH-1. Junction PCR analysis confirms precise donor integration by using the primer sets spanning transgene and host genome outside homology arms [53]. Primer sets spanning left-hand side homologous sequence (LHS) and right-hand side homologous sequence (RHS) were used to perform 5′ junction PCR (Figure 2(e)) and 3′ junction PCR (Figure 2(f)), respectively. According to our results, a linear (cut) donor with 1 : 1 and 1 : 3 molar ratios of Cas9/gRNA to the donor yielded a successful knock-in in unsynchronized cells which represents code 6 and 3, respectively. Similarly, a circular (uncut) donor yielded a knock-in at a 1 : 3 molar ratio of Cas9/gRNA to the donor in cell cycle–synchronized cells (code 4). Targeted integration was not observed in remaining combinations with nCas9 (codes 5, 7, and 8). We also did not find targeted integration of donor by wtCas9/gRNA1 in the presence of both circular (code 1) and linear (code 2) configurations of donor plasmid (Figures 2(d)–2(f)). Overall, 3 out of 8 (37.5%) blasticidin-resistant HEK293T clones were positive for targeted integration of donor at CASH-1 site. Based upon the results with HEK293T, HDFs were transfected in 8 different combinations with nCas9 only (Figure 2(g)). Out of them, only 2 (25%) combinations allowed transfected HDFs to survive under blasticidin-selection (represented as codes 1 and 2) unlike others (“-” sign; Figure 2(g)). Both of these survived clones of HDFs were confirmed by junction PCRs for targeted integration of donor at CASH-1 locus (Figure 2(h)). We noted that the circular and linearized donor at the 1 : 1 (code 1) and 1 : 3 (code 2) molar ratio of Cas9/gRNA to the donor was effective in unsynchronized cells, respectively (Figures 2(g) and 2(h)). The remaining combinations failed to integrate donor cassette at CASH-1 site of HDFs. In case of HEK293T, cells obtained under numerical code 4 were used for further experiments due to the absence of nonspecific amplicons (Figures 2(e) and 2(f)). Clones of HEK293T cells and HDFs with right integration of donor cassette at CASH-1 were named as HEK293T-OSK and HDF-OSK, respectively. Despite lower integration efficiency of CRISPR/Cas9 tool for larger transgenes via homology-directed repair pathway [54, 55], we successfully optimized the integration of reprogramming cassette (transgene) at CASH-1 site.

Clones with targeted integration of transgene were enriched by performing flow cytometry sorting for DOX-treated HEK293T-OSK (Supplementary Figure 5(a)) and HDF-OSK cells (Supplementary Figure 5(b)). We observed heterogeneous expression of GFP in a homogenous population of cells which could be possibly due to the noise in gene expression [56, 57]. Cells with higher expression of GFP were sorted and expanded in growth medium. DOX-induced expression of reprogramming factors was reconfirmed in sorted cells by RT-PCR analysis of *OCT4*, *SOX2*, and *KLF4* transcripts in the total mRNA isolated from induced HEK293T-OSK (Figure 2(i)) and HDF-OSK cells (Figure 2(j)). We noticed a GFP signal in the sorted cells only upon DOX induction, thus confirming the induced expressivity of the donor cassette even after integration at CASH-1 (Figure 2(k)).

### 3.3. Proliferative and Immunological Validation

The proliferative validation of cells engineered at CASH-1 GSH was done by analyzing their viability, while their immunological response was validated by detecting the level of secreted cytokines after the treatment of ligands of various immune signaling pathways. The responses of wild-type (HEK293T and HDF) and genetically modified stable cell lines (HEK293T-OSK and HDF-OSK) in the presence or absence of DOX as well as immunostimulatory ligands were compared using three-way ANOVA for differences with the variables of (a) cell type; (b) ligand treatment; and (c) DOX treatment. For viability without DOX treatment, we did not observe statistically significant difference in the optical density between HEK293T and HEK293T-OSK cells in both ligand-treated and untreated groups. Similarly, no significant difference was observed among DOX-treated groups ((I) in Figure 3(a)). However, each DOX-treated group showed a significant difference (∗∗∗) in the optical density with that of untreated group ((II) in Figure 3(a)). For viability of HDF and HDF-OSK, we did not observe any significant difference in the optical density among all three variables (Figure 3(b)). We also analyzed the secretion pattern of IL-6 by the cells treated as mentioned above. In terms of IL-6 secretion by HDF and HDF-OSK, we did not observe any significant difference among all three variables within each ligand group ((I) and (II) in Figure 3(c)). However, significant secretion of IL-6 (∗∗∗) was observed for all variables in comparison to their respective controls after treating them with ligands of various signaling pathways ((III) in Figure 3(c)). We did not observe the secretion of IL-6 by the HEK293T and HEK293T-OSK (data not shown).

### 3.4. Generation and Characterization of iPSCs

**Figure 4:**
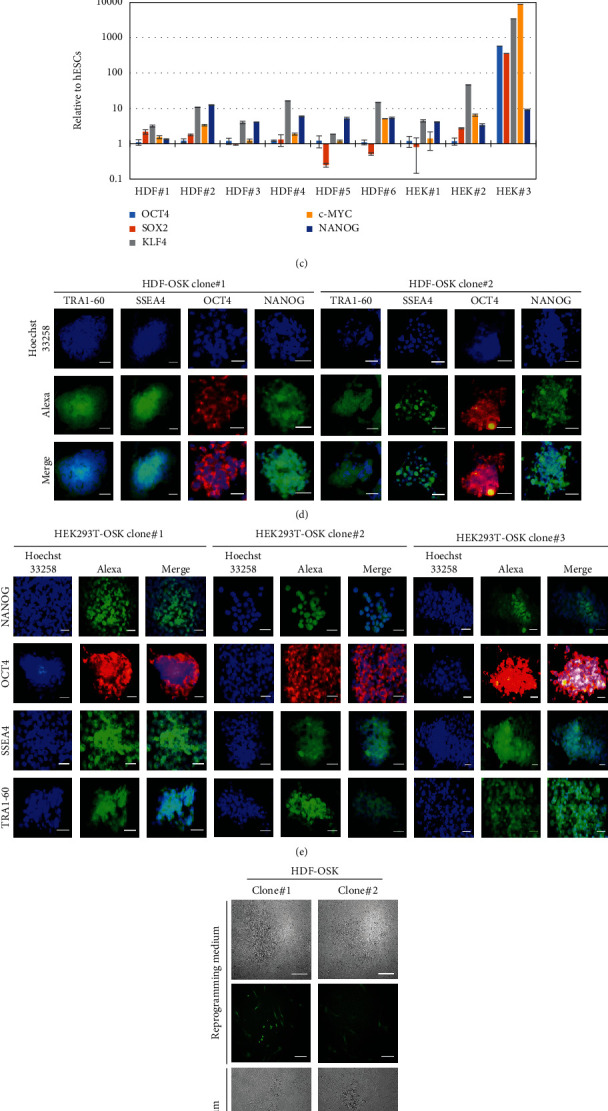
Generation and characterization of the iPSCs. (a) Workflow for the generation of iPSCs from HDF-OSK and HEK293T-OSK cells using a reprogramming medium. (b) Morphological changes during the reprogramming of HDF-OSK and HEK293T-OSK cells into iPSCs followed by their expansion in a maintenance medium. Scale bar: 100 *μ*m. (c) Expression levels of pluripotency markers in the generated iPSC clones in comparison with human embryonic stem cells (hESCs). (d and e) Immunofluorescence assays of pluripotency markers in the iPSC clones derived from HDF-OSK cells (d) or HEK293T-OSK cells (e). Scale bar: 50 *μ*m. (f) Silencing of GFP expression in morphologically changed HDF-OSK clones observed by fluorescence microscopy. Scale bar: 200 *μ*m.

Each individual cell of iPSCs shows round shape with scant cytoplasm and large nucleolus. They form sharp-edged, tightly packed, flat colonies similar to human embryonic stem cells (hESCs). HDF-OSK and HEK293T-OSK cells (9 × 10^4^/well) were subjected to the schematic process of iPSC generation in 6-well plate (Figure 4(a)). After 12 days of reprogramming, we observed around 10 and 15 morphologically changed tightly packed iPSC clones from HDF-OSK and HEK293T-OSK cells, respectively, which grew in size for further 12 days. It indicates the reprogramming efficiency around 0.011 ± 0.001 (*n* = 3) and 0.018 ± 0.002 (*n* = 3) for HDF-OSK and HEK293T-OSK cells, respectively. These clones were manually picked and transferred to a Matrigel-coated dish containing a stem cell maintenance medium (Figure 4(b)). We picked six and three clones derived from HDF-OSK (HDF#1-6) and HEK293T-OSK (HEK#1-3), respectively, and found their mRNA expression of pluripotency markers comparable to a positive control, human embryonic stem cells (Figure 4(c)) [58, 59]. We further confirmed pluripotency markers by immunofluorescence staining of two HDF-OSK clones (clone#1 and 2; Figure 4(d)) and three HEK293T-OSK clones (clone#1-3; Figure 4(e)). On the other hand, we could not detect signal for any marker in HDFs (Supplementary Figure 6(a)) and HEK293T (Supplementary Figure 6(b)) as negative controls. The FACS analysis further confirmed the proportion of HDF-OSK and HEK293T-OSK iPSC clones expressing SSEA-4 (10.4% to 20.5%) and TRA1-60 (3% to 20.8%) pluripotency markers in total cell population (Supplementary Figure 7). Generation of a fully reprogrammed iPSC is associated with the silencing of the transgene [60]. Our reprogramming transgene contains GFP as an expression marker whose expression was not detected in our generated representative iPSC clones under fluorescence microscopy (Figure 4(f)).

**Figure 5:**
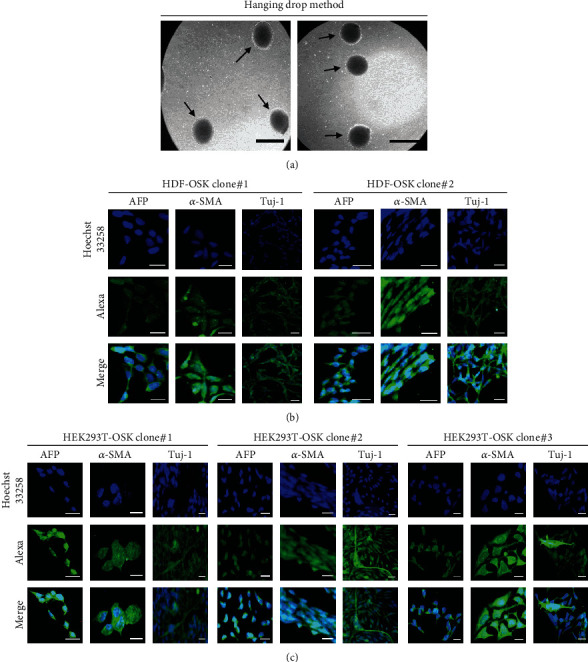
The differentiation potential of iPSCs. (a) Formation of embryoid bodies (black arrowhead) by the hanging drop method. Scale bar: 100 *μ*m. (b and c) Immunofluorescence staining of spontaneously differentiating embryoid bodies of HDF-OSK (b) or HEK293T-OSK (c) clones for markers of each germ layer, i.e., *α*-fetoprotein (AFP) for endoderm, *α*-smooth muscle actin (*α*-SMA) for mesoderm, and *β* III tubulin (TUJ-1) for ectoderm. Nuclei were stained with a Hoechst 33258 solution, and images were captured by means of a fluorescence microscope (Olympus IX53; Olympus Corporation, Tokyo, Japan). Scale bar: 25 *μ*m.

Pluripotency of generated iPSC clones was confirmed by their ability to form embryoid bodies (Figure 5(a)) and spontaneously differentiate into derivatives of each germ layer. The clones from HDF-OSK (clones#1 and 2; Figure 5(b)) and HEK-OSK (clone#1-3; Figure 5(c)) were tested positive for differentiation markers of all three germ layers, while no such markers were identified in negative controls including HDF (Supplementary Figure 8(a)) and HEK293T (Supplementary Figure 8(b)).

## 4. Discussion

The reliable expression of genome-integrated transgenes is beneficial for studying function of genes as well as lineage analysis by using certain reporter systems. It emphasizes on inserting transgene at a suitable location (GSHs) on the human genome. Among GSHs, adeno-associated virus site 1 (AAVS1) [22, 23], chemokine (C-C motif) receptor 5 (CCR5) [24], and ROSA26 [25] are being extensively used [26, 27]. Previously, human foreskin fibroblasts (HFFs) were reprogrammed by integrating reprogramming genes into AAVS1 locus through zinc-finger nuclease technology [61]. Gaucher's disease and cystic fibrosis-specific iPSCs have been generated from patient fibroblasts by integrating reprogramming factors at CCR5 locus by transcription activator-like effector nucleases [62]. ROSA26 locus has also been used to provoke reprogramming of mouse somatic cells [63, 64]. The CASH-1 GSH was first time reported by targeting bone marrow MSCs and skin fibroblasts with the nonspecific lentiviral system [28]. Here, we exploited CASH-1 locus in HDFs as well as HEK293T cells in order to reprogram them into iPSCs by integrating reprogramming cassette with more precise CRISPR/Cas9 genome editing tool. One limitation of our study is to get iPSCs with footprints of transgene; however, their presence in GSH might not put severe deleterious effects. Alternatively, footprint-free iPSCs can be generated by methods involving Sendai virus [65], adenovirus [66], piggyBac system [67], episomal vectors [68], minicircle vector [69], synthetic mRNA [70], or direct protein delivery [71]. However, certain measures must be taken to enhance transgene expression and minimize host-immune response during some of these methods [72, 73].

The progression of a regular cell cycle is based upon associated cyclins. Their expression is controlled by cellular transcription factors such as NF-*κ*B and AP-1 which are activated by the activation of various cellular receptors [29–35]. By our study, we did not observe change in cell viability of HEK293T-OSK and HDF-OSK cells as compared to their nonengineered parental cells. We observed lesser growth of DOX-treated HEK293T-OSK and HEK293T cells as compared to those without DOX-treatment; however, it was not the case with HDF-OSK and HDF. This could be because of the changes in the metabolism of cancerous cell lines with the treatment of DOX [74–76]. To examine immunological response, we analyzed the pattern of IL-6 secretion by HDF-OSK in comparison to parental cells, HDFs. The IL-6 secretion level was same between both types of cells.

The iPSCs have been generated from multiple target cells including mouse fibroblasts [77], human fibroblasts [61, 78], human keratinocytes [79], human peripheral blood cells [80], and renal epithelial cells [81]. The generation of iPSCs from primary (HDFs) as well as cancerous cells (HEK293T) by using the current approach supports its reproducibility and broader-applicability. In order to understand the mechanism of cancer progression [82, 83], reprogramming has been done for multiple cancerous cell lines including CHLA-10 [84], SH-IN [85], MCF-7 [86], A549 [87], and HEK293 [88]. Our strategy can be adopted to reprogram cancerous cell line (for instance, HEK293T) into iPSCs. The reprogramming process can be deleterious with the inclusion of oncogene *c-MYC* in the reprogramming factors [77] or with the uncontrolled expression of reprogramming factors [89, 90]. We overcome these factors by eliminating the *c-MYC* gene and controlling the expression with doxycycline induction, respectively. The constant expression of transcription factors present in the reprogramming cassette may interfere with the reprogramming process or functional properties of cells obtained from the differentiation of iPS cells [91–93]. Despite drug-inducible reprogramming cassette, there are chances for the leaky expression of reprogramming factors at the stage of differentiation [94]. Our reprogramming cassette is flanked with two loxP sites which can be used to excise cassette by using Cre/loxP system before any clinical applications [95, 96]. Future work will show whether (i) this approach can generate iPSCs from other cell types such as keratinocytes, human peripheral blood cells, and renal epithelial cells [79–81]; and (ii) the efficiency of iPSC generation can be increased by using some enhancing factors [97–100]; adjusting cell numbers [101]; or optimizing DOX-treatment [102, 103].

## 5. Conclusions

Collectively, our study provides an alternative approach to make iPSCs by targeting the CASH-1 with the help of CRISPR/Cas9 tool. Moreover, it also confirms processes such as proliferation and ability to respond to ligands of various cellular signaling pathways. It signifies the usage of CASH-1 site for multiple therapeutic as well as biotechnological purposes by inserting any gene of interest.

## Data Availability

The data used to support the findings of this study are included within the article and supplementary information file.

## Supplementary Materials

Supplementary Materials Supplementary Figure 1: the CASH-1 sequence and target sites. The shown sequence of chromosome 1 constitutes positions 188,082,217–188,083,803, where the CASH-1 site (188,083,272) is underlined. The sites for designing gRNAs are boldfaced, with PAM sequences (5′-NGG-3′) in red font. The target sites for the first and second primer set required for the T7 endonuclease I assay are highlighted in yellow and green, respectively. Supplementary Figure 2: cloning and sequencing confirmation of designed gRNAs. (a) Colony PCR confirmation of the gRNAs cloned into pX330-U6-Chimeric_BB-CBh-hSpCas9. (b) Sanger sequencing confirmation of the gRNAs cloned into pX330-U6-Chimeric_BB-CBh-hSpCas9. (c) Colony PCR confirmation of the gRNAs cloned into pX335-U6-Chimeric_BB-CBh-hSpCas9n(D10A). (d) Sanger sequencing confirmation of the gRNAs cloned into pX335-U6-Chimeric_BB-CBh-hSpCas9n(D10A). In colony PCR (a, c), each number indicates the relevant gRNA; a capitalized letter denotes a colony name; and lowercase “n” represents pX335-U6-Chimeric_BB-CBh-hSpCas9n(D10A). L: 100 bp ladder. Supplementary Figure 3: the design and sequence of the reprogramming-donor cassette. The design (above) and DNA sequence (below) of the polycistronic DOX-inducible expression cassette is shown; each component is named and labeled with a different color. The red arrows under the cassette represent restriction sites, and the cassette is flanked by sequences homologous to CASH-1. Abbreviations: LHS: left-hand homologous sequence; SV40 poly A: simian virus 40 polyadenylation signal; mPGK promoter: mouse phosphoglycerate kinase promoter; TetO: tetracycline operator; OCT4: octamer-binding transcription factor 4; SOX2: sex-determining region Y-box 2; KLF4: Krüppel-like factor 4; EGFP: enhanced green fluorescent protein; bGH poly A: bovine growth hormone (bGH) poly A signal; BSD: blasticidin; RHS: right-hand homologous sequence. Supplementary Figure 4: optimizing concentration of DOX. Fluorescence imaging of donor-transfected HEK293T cells for GFP marker after 48 h induction with various concentrations (1-5 *μ*g/ml) of DOX. Scale bar: 500 *μ*m. Supplementary Figure 5. Enrichment of GFP-positive cells. (a and b) Sorting of DOX-induced HEK293T-OSK cells (a) and HDF-OSK cells (b) on the basis of GFP signal intensity during flow cytometry (FACSAria III, BD Biosciences). The data processing was done in the FACSDiva software (BD Biosciences). Supplementary Figure 6: analysis of pluripotency markers in negative controls. (a and b) Immunofluorescence analysis of pluripotency markers in HDFs (a) and HEK293T (b). Scale bar: 100 *μ*m. Supplementary Figure 7: FACS analysis of cell surface pluripotency markers. Indirect flow cytometry analysis of iPSC clones stained with SSEA-4 or TRA1-60 specific primary antibodies and Alexa Fluor 488– or Alexa Fluor 647–conjugated secondary antibodies, respectively. The analysis was done in FACSAria III, BD Biosciences instrument and data processing was done in the FACSDiva software (BD Biosciences). Supplementary Figure 8: analysis of germ-layer markers in negative controls. (a and b) Immunofluorescence analysis of germ-layer markers in HDFs (a) and HEK293T (b). Scale bar: 50 *μ*m. Supplementary Table 1: list of predicted off-targets. Supplementary Table 2: CRISPR RNA (crRNA) oligonucleotides specific to target sites throughout CASH-1. Supplementary Table 3: oligonucleotides used in this study.
