# Supplementary material for: An Alternate Approach to Generate Induced Pluripotent Stem Cells with Precise CRISPR/Cas9 Tool
Source: Stem Cells Int. 2022 Sep 22;2022:4537335. doi: 10.1155/2022/4537335 (PMC9522500; doi:10.1155/2022/4537335)
Supplement: Supplementary Materials — Supplementary Figure 1: the CASH-1 sequence and target sites. The shown sequence of chromosome 1 constitutes positions 188,082,217–188,083,803, where the CASH-1 site (188,083,272) is underlined. The sites for designing gRNAs are boldfaced, with PAM sequences (5′-NGG-3′) in red font. The target sites for the first and second primer set required for the T7 endonuclease I assay are highlighted in yellow and green, respectively. Supplementary Figure 2: cloning and sequencing confirmation of designed gRNAs. (a) Colony PCR confirmation of the gRNAs cloned into pX330-U6-Chimeric_BB-CBh-hSpCas9. (b) Sanger sequencing confirmation of the gRNAs cloned into pX330-U6-Chimeric_BB-CBh-hSpCas9. (c) Colony PCR confirmation of the gRNAs cloned into pX335-U6-Chimeric_BB-CBh-hSpCas9n(D10A). (d) Sanger sequencing confirmation of the gRNAs cloned into pX335-U6-Chimeric_BB-CBh-hSpCas9n(D10A). In colony PCR (a, c), each number indicates the relevant gRNA; a capitalized letter denotes a colony name; and lowercase “n” represents pX335-U6-Chimeric_BB-CBh-hSpCas9n(D10A). L: 100 bp ladder. Supplementary Figure 3: the design and sequence of the reprogramming-donor cassette. The design (above) and DNA sequence (below) of the polycistronic DOX-inducible expression cassette is shown; each component is named and labeled with a different color. The red arrows under the cassette represent restriction sites, and the cassette is flanked by sequences homologous to CASH-1. Abbreviations: LHS: left-hand homologous sequence; SV40 poly A: simian virus 40 polyadenylation signal; mPGK promoter: mouse phosphoglycerate kinase promoter; TetO: tetracycline operator; OCT4: octamer-binding transcription factor 4; SOX2: sex-determining region Y-box 2; KLF4: Krüppel-like factor 4; EGFP: enhanced green fluorescent protein; bGH poly A: bovine growth hormone (bGH) poly A signal; BSD: blasticidin; RHS: right-hand homologous sequence. Supplementary Figure 4: optimizing concentration of DOX. Fluorescence imaging of donor-t [file 4537335.f1.docx]

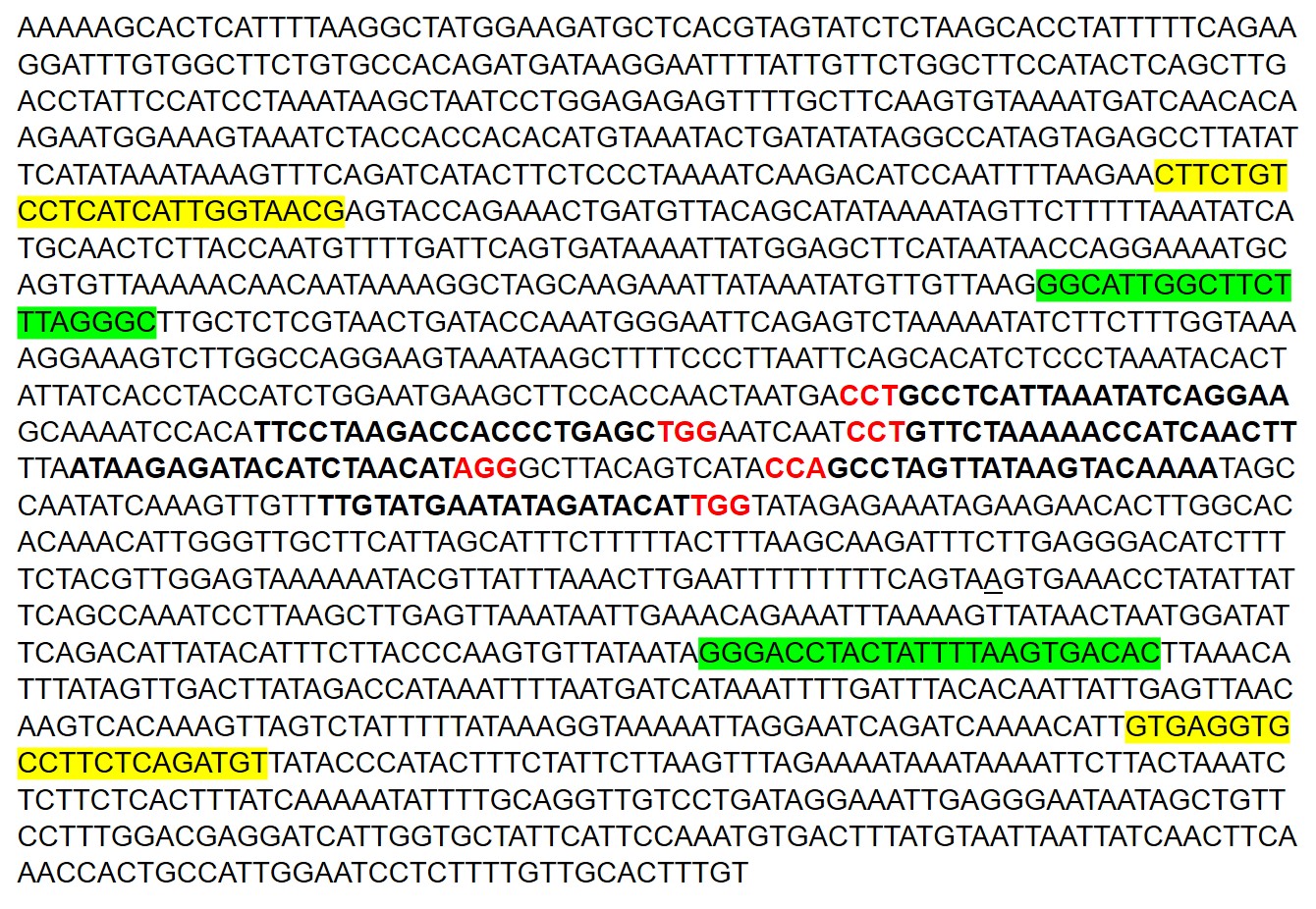


Supplementary Figure 1. The CASH-1 sequence and target sites. The shown sequence of chromosome 1 constitutes positions 188,082,217–188,083,803, where the CASH-1 site (188,083,272) is underlined. The sites for designing gRNAs are boldfaced, with PAM sequences (5ʹ-NGG-3ʹ) in red font. The target sites for the first and second primer set required for the T7 endonuclease I assay are highlighted in yellow and green, respectively.


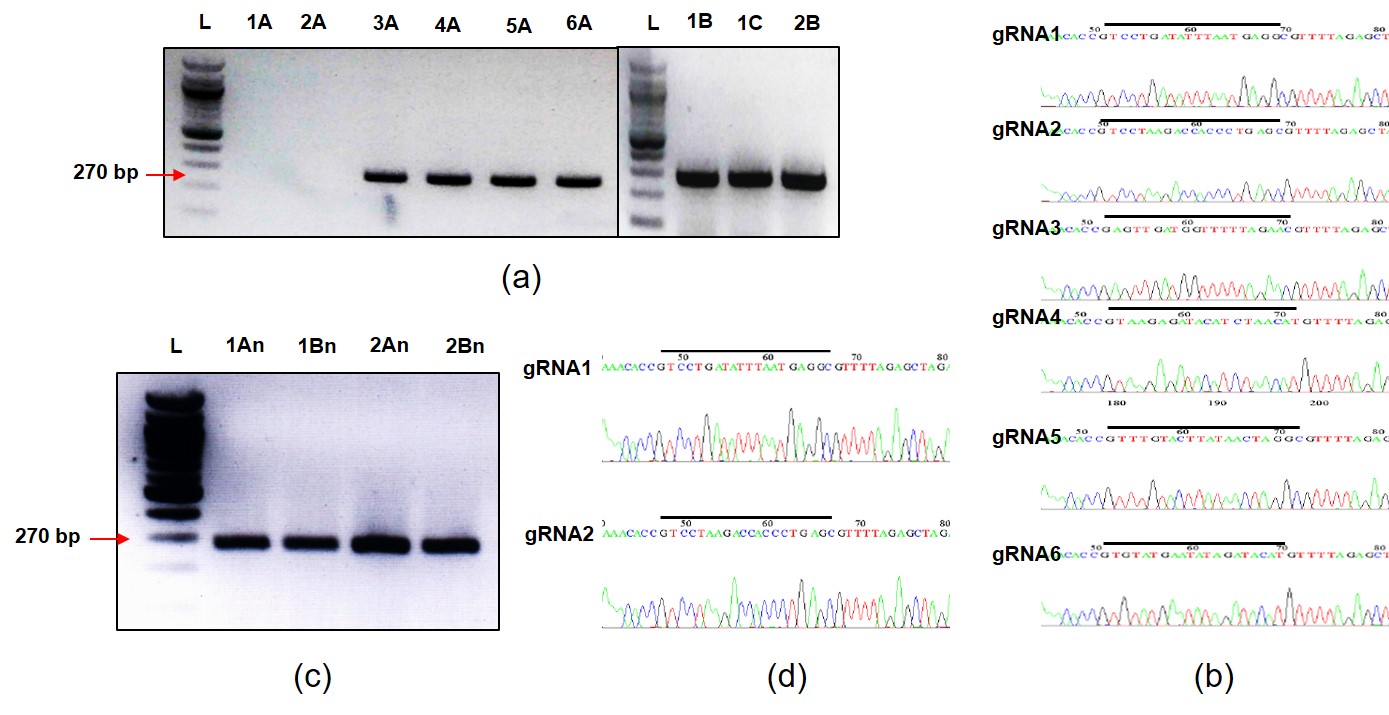


Supplementary Figure 2. Cloning and sequencing confirmation of designed gRNAs. (a) Colony PCR confirmation of the gRNAs cloned into pX330-U6-Chimeric_BB-CBh-hSpCas9. (b) Sanger sequencing confirmation of the gRNAs cloned into pX330-U6-Chimeric_BB-CBh-hSpCas9. (c) Colony PCR confirmation of the gRNAs cloned into pX335-U6-Chimeric_BB-CBh-hSpCas9n(D10A). (d) Sanger sequencing confirmation of the gRNAs cloned into pX335-U6-Chimeric_BB-CBh-hSpCas9n(D10A). In colony PCR (a, c), each number indicates the relevant gRNA; a capitalized letter denotes a colony name; and lowercase “n” represents pX335-U6-Chimeric_BB-CBh-hSpCas9n(D10A). L: 100 bp ladder.


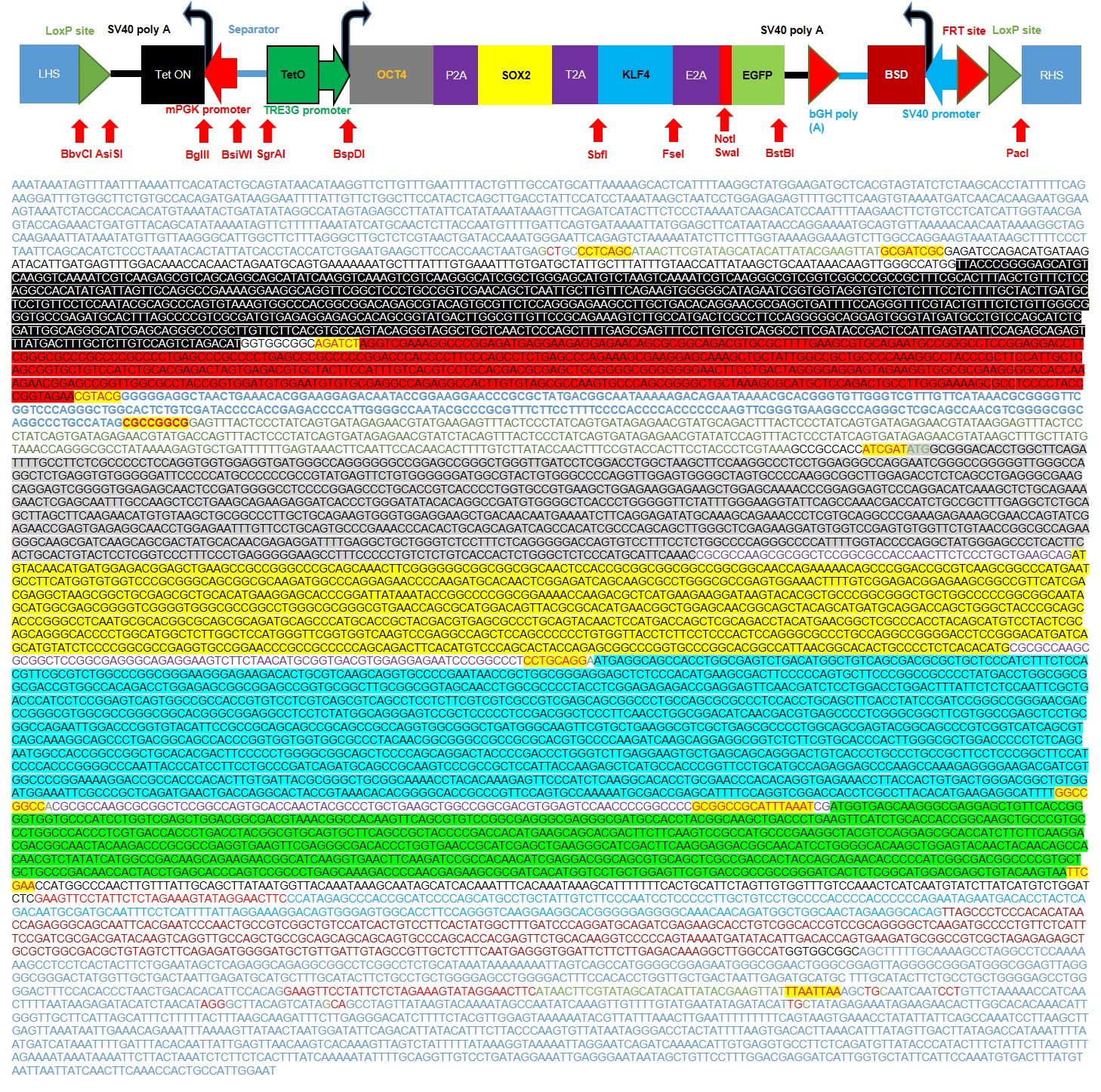


Supplementary Figure 3. The design and sequence of the reprogramming-donor cassette. The design (above) and DNA sequence (below) of the polycistronic DOX-inducible expression cassette is shown; each component is named and labeled with a different color. The red arrows under the cassette represent restriction sites, and the cassette is flanked by sequences homologous to CASH-1. Abbreviations: LHS, left-hand homologous sequence; SV40 poly A, simian virus 40 polyadenylation signal; mPGK promoter, mouse phosphoglycerate kinase promoter; TetO, tetracycline operator; OCT4, octamer-binding transcription factor 4; SOX2, sex-determining region Y-box 2; KLF4, Krüppel-like factor 4; EGFP, enhanced green fluorescent protein; bGH poly A, bovine growth hormone (bGH) poly A signal; BSD, blasticidin; RHS, right-hand homologous sequence.


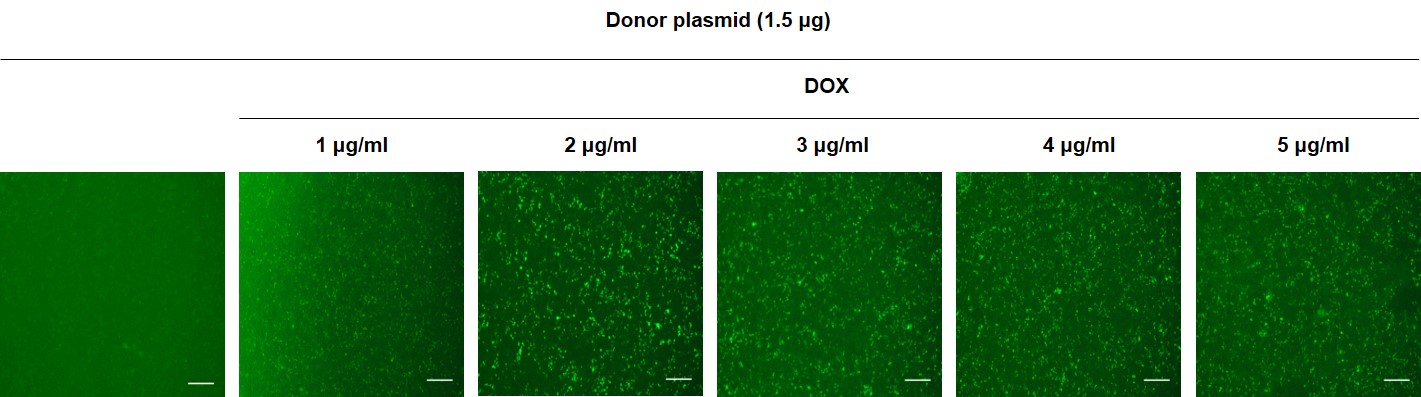


Supplementary Figure 4. Optimizing concentration of DOX. Fluorescence imaging of donor-transfected HEK293T cells for GFP marker after 48 h induction with various concentrations (1-5 μg/ml) of DOX. Scale bar: 500 μm.


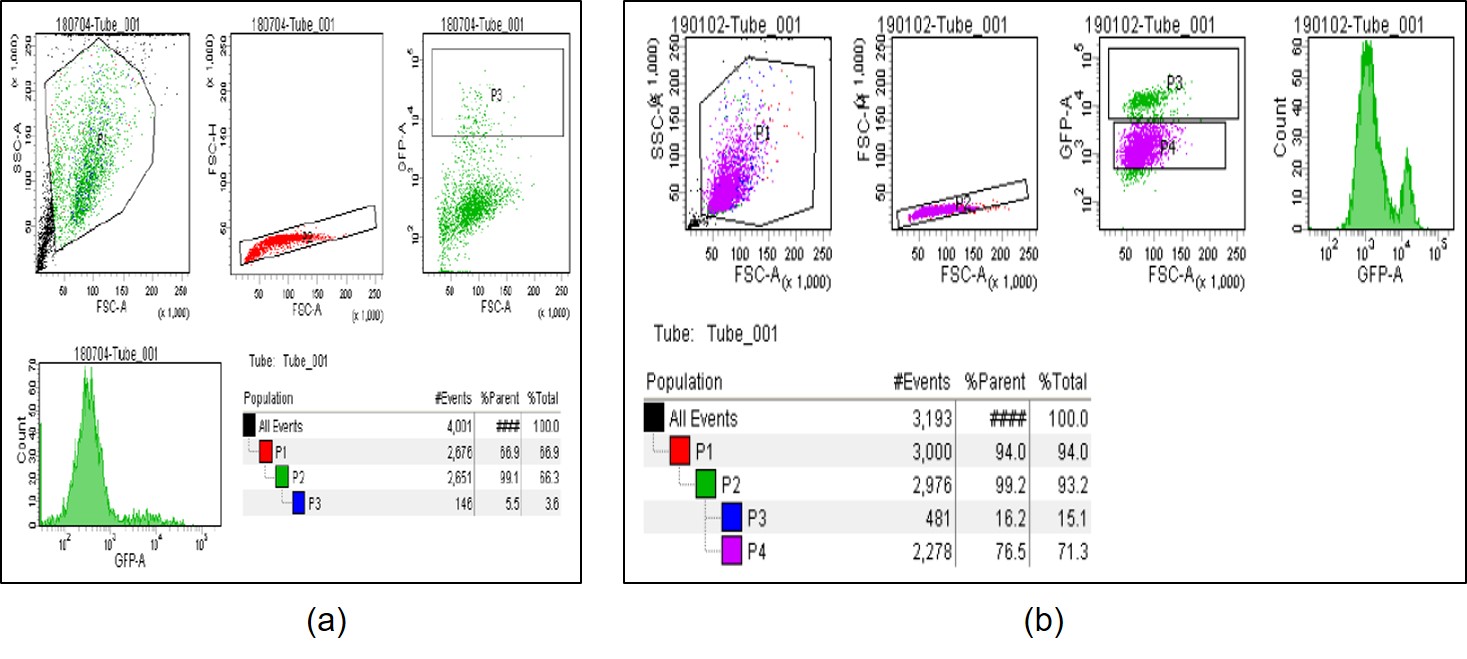


Supplementary Figure 5. Enrichment of GFP-positive cells. (a, b) Sorting of DOX-induced HEK293T-OSK cells (a) and HDF-OSK cells (b) on the basis of GFP signal intensity during flow cytometry (FACSAria III, BD Biosciences). The data processing was done in the FACSDiva software (BD Biosciences).


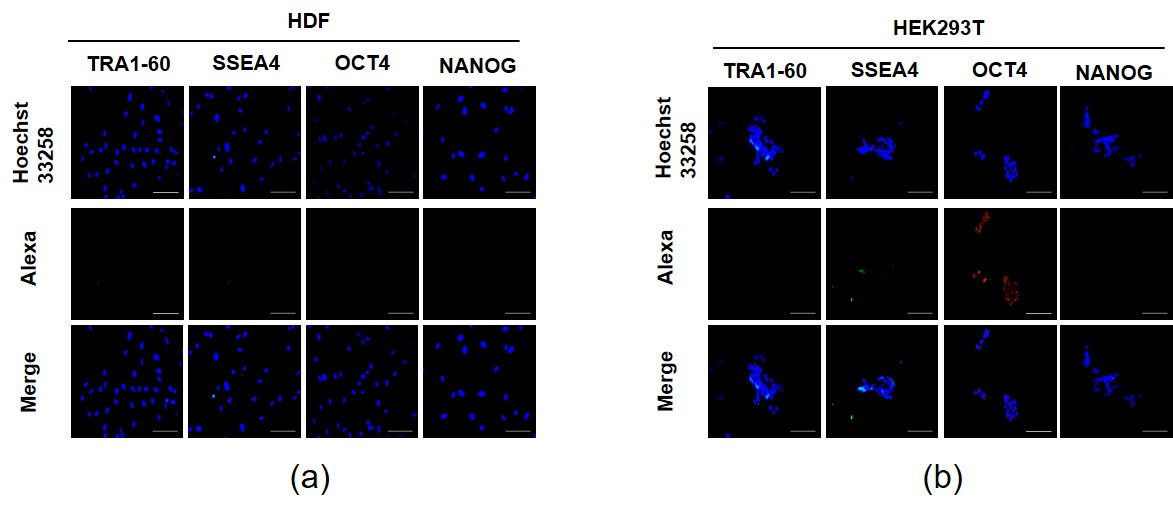


Supplementary Figure 6. Analysis of pluripotency markers in negative controls. (a, b) Immunofluorescence analysis of pluripotency markers in HDFs (a) and HEK293T (b). Scale bar: 100 μm.


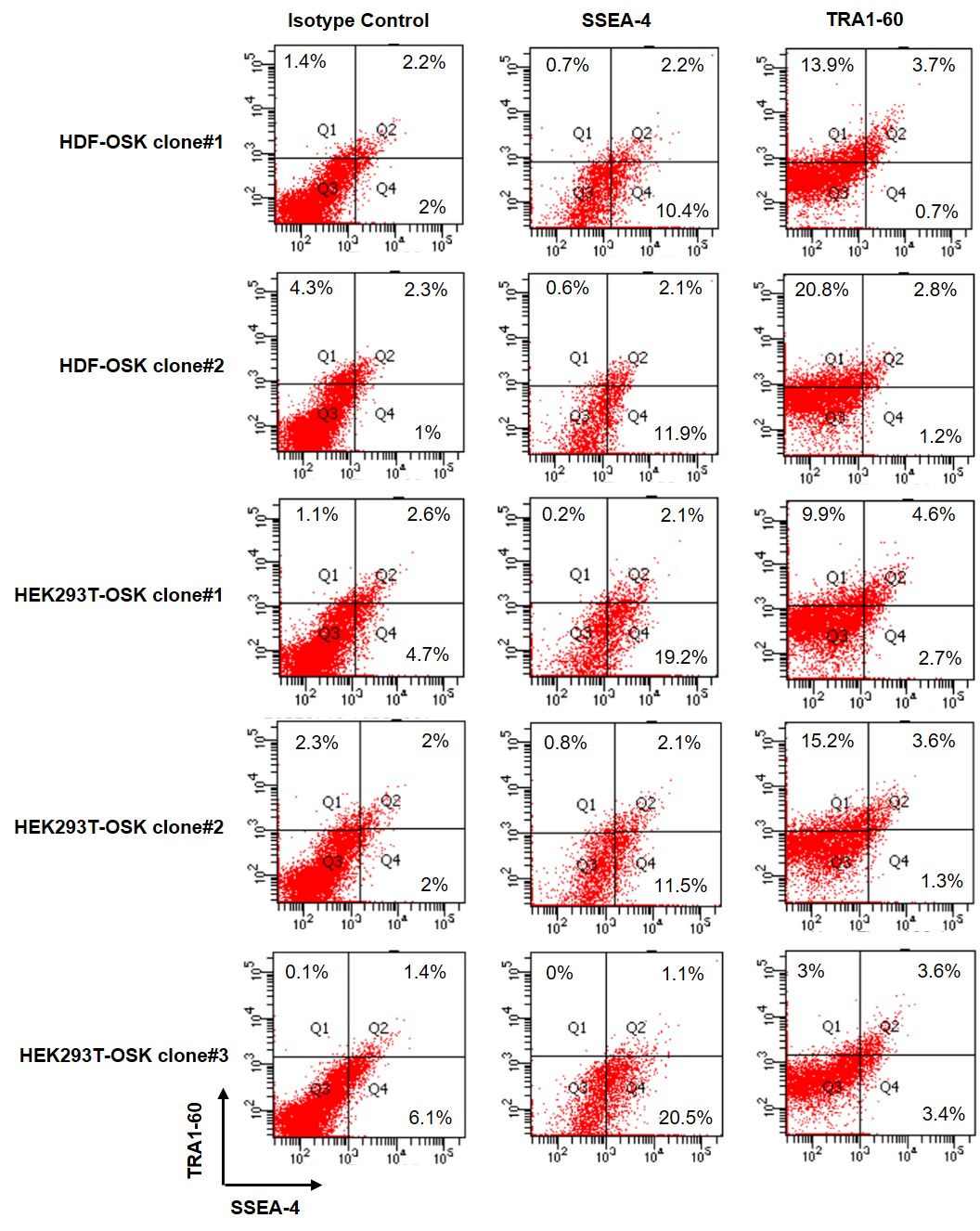


Supplementary Figure 7. FACS analysis of cell surface pluripotency markers. Indirect flow cytometry analysis of iPSC clones stained with SSEA-4 or TRA1-60 specific primary antibodies and Alexa Fluor 488– or Alexa Fluor 647–conjugated secondary antibodies, respectively. The analysis was done in FACSAria III, BD Biosciences instrument and data processing was done in the FACSDiva software (BD Biosciences).


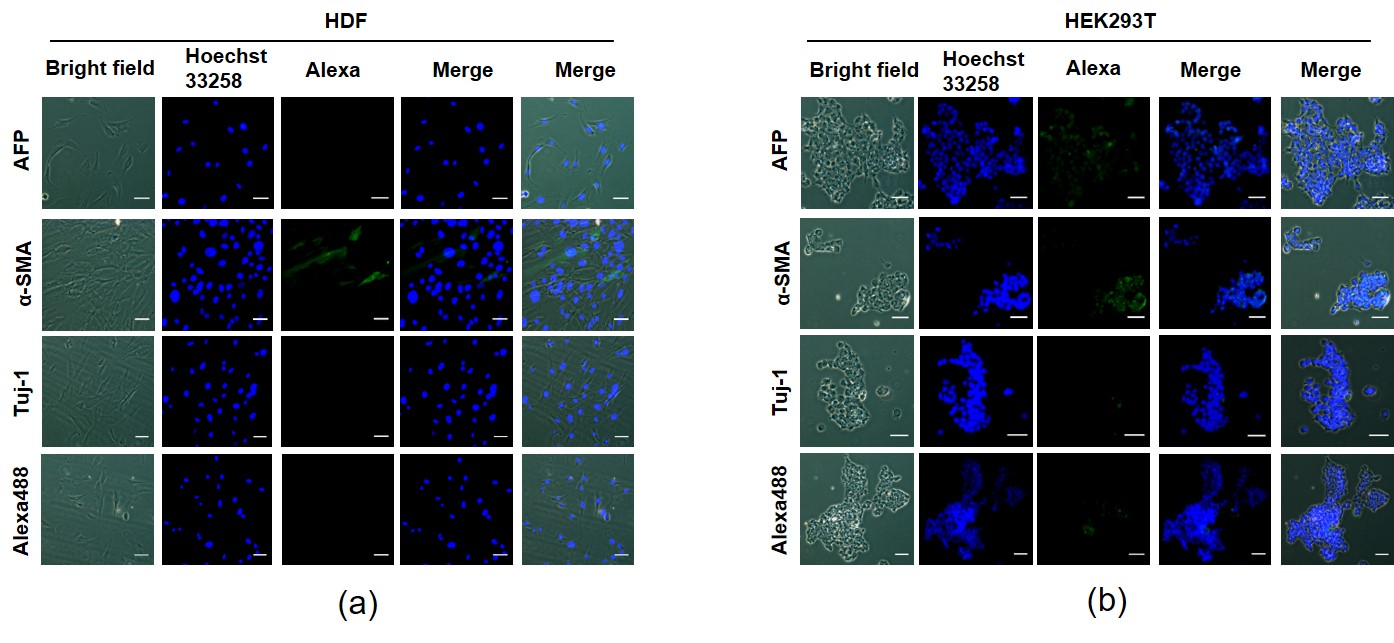


Supplementary Figure 8. Analysis of germ-layer markers in negative controls. (a, b) Immunofluorescence analysis of germ-layer markers in HDFs (a) and HEK293T (b). Scale bar: 50 μm.

Supplementary Table 1. List of predicted off-targets.

| **crRNA** | **Target Sequence** | **Chromosome** | **Position** | **Direction** | **Mismatches** | **T7E1 amplicon** |
| --- | --- | --- | --- | --- | --- | --- |
| GCCTCATTAAATATCAGGAANGG | GCCTCATTAAAaATCAGGttTGG | chr15 | 48169892 | - | 3 |  |
| TTCCTAAGACCACCCTGAGCNGG | TcCCTcAGACCACCCTGAaCAGG | chr5 | 78547839 | - | 3 |  |
| TTCCTAAGACCACCCTGAGCNGG | TTCCTAAGAtCACCtgGAGCAGG | chr5 | 146343605 | + | 3 |  |
| TTCCTAAGACCACCCTGAGCNGG | TTCCTAtGgCCcCCCTGAGCTGG | chr5 | 158027812 | - | 3 |  |
| GCCTCATTAAATATCAGGAANGG | GCCTgcTTAAATATtAGGAATGG | chr20 | 46879054 | - | 3 |  |
| TTCCTAAGACCACCCTGAGCNGG | gTgCTAAGAgCACCCTGAGCTGG | chr20 | 17732150 | - | 3 |  |
| TTCCTAAGACCACCCTGAGCNGG | cTCCTAAcACCACCCTGAaCGGG | chr1 | 29739097 | + | 3 |  |
| TTCCTAAGACCACCCTGAGCNGG | TTCCTAAGACCACCCTGAGCTGG | chr1 | 188082965 | + | 0 | Yes |
| TTCCTAAGACCACCCTGAGCNGG | TcCCTAAGgCCACaCTGAGCAGG | chr22 | 40950742 | - | 3 |  |
| GCCTCATTAAATATCAGGAANGG | GCaTCATTAAATATCAtGAcAGG | chr2 | 219864544 | - | 3 |  |
| TTCCTAAGACCACCCTGAGCNGG | TTtCTAAGtCCACCCTGgGCTGG | chr2 | 219426500 | - | 3 |  |
| TTCCTAAGACCACCCTGAGCNGG | TgCCTgAGACCACCCaGAGCTGG | chr2 | 235286083 | + | 3 |  |
| GCCTCATTAAATATCAGGAANGG | GCaTCATaAAATATgAGGAATGG | chr4 | 33237293 | + | 3 |  |
| TTCCTAAGACCACCCTGAGCNGG | TTgCcAgGACCACCCTGAGCTGG | chr9 | 95693279 | - | 3 |  |
| TTCCTAAGACCACCCTGAGCNGG | TTCCTAAGACCcCCtTGgGCTGG | chr6 | 40251153 | + | 3 |  |
| TTCCTAAGACCACCCTGAGCNGG | TTCtTAgGACCAtCCTGAGCAGG | chr11 | 45888069 | - | 3 |  |
| GCCTCATTAAATATCAGGAANGG | GCCTaATTAAATAgCAGGAAGGG | chr10 | 103281077 | + | 2 |  |
| TTCCTAAGACCACCCTGAGCNGG | TgCtgAAGACCACCCTGAGCTGG | chr10 | 13929320 | + | 3 |  |
| TTCCTAAGACCACCCTGAGCNGG | TTCCTAAaACaACCCTGAGCGGG | chr19 | 29204049 | + | 2 |  |
| TTCCTAAGACCACCCTGAGCNGG | aTCCTAAGACCACCtTGgGCTGG | chr3 | 59679444 | - | 3 |  |

Supplementary Table 2. CRISPR RNA (crRNA) oligonucleotides specific to target sites throughout CASH-1.

| **Name** | **Target Site sequence (5’-3’)** | **Direction** | **5’-Sequence-3’** |
| --- | --- | --- | --- |
| g1 | **CCT**GCCTCATTAAATATCAGGAA | F | CACCGTCCTGATATTTAATGAGGC |
|  |  | R | AAACGCCTCATTAAATATCAGGAC |
| g2 | TTCCTAAGACCACCCTGAGC**TGG** | F | CACCGTCCTAAGACCACCCTGAGC |
|  |  | R | AAACGCTCAGGGTGGTCTTAGGAC |
| g3 | **CCT**GTTCTAAAAACCATCAACTT | F | CACCGAGTTGATGGTTTTTAGAAC |
|  |  | R | AAACGTTCTAAAAACCATCAACTC |
| g4 | ATAAGAGATACATCTAACAT**AGG** | F | CACCGTAAGAGATACATCTAACAT |
|  |  | R | AAACATGTTAGATGTATCTCTTAC |
| g5 | **CCA**GCCTAGTTATAAGTACAAAA | F | CACCGTTTGTACTTATAACTAGGC |
|  |  | R | AAACGCCTAGTTATAAGTACAAAC |
| g6 | TTGTATGAATATAGATACAT**TGG** | F | CACCGTGTATGAATATAGATACAT |
|  |  | R | AAACATGTATCTATATTCATACAC |

Supplementary Table 3. Oligonucleotides used in this study.

| **Primers for colony PCR and sequencing** | | | |
| --- | --- | --- | --- |
| Name | Direction | 5’-sequence-3’ | Amplicon size (bp) |
| U6 promoter | F | GAGGGCCTATTTCCCATG | 270 |
| **Primers for T7E1 assay** | | | |
| 1^st^ primer set | F | CTTCTGTCCTCATCATTGGTAACG | 981 |
|  | R | ACATCTGAGAAGGCACCTCAC |  |
| 2^nd^ primer set | F | GGCATTGGCTTCTTTAGGGC | 628 |
|  | R | GTGTCACTTAAAATAGTAGGTCCC |  |
| **Primers for junction PCR** | | | |
| 5’ Junction PCR | Outer-FP | TCCAATAAAATAAGCGGGTGAC | 3081 |
|  | Donor-RP2 | GGCGCCCTGGTTTACATAAG |  |
| 3’ Junction PCR | Donor-FP6 | GTTCCCATCTCAAGGCACAC | 3126 |
|  | Outer-RP | TCCACCATTCTCCCACTGAC |  |
| **Primers for RT-PCR** | | | |
| *OCT4* | F | TTGGGCTCGAGAAGGATGTG | 91 |
|  | R | TCCTCTCGTTGTGCATAGTCG |  |
| *SOX2* | F | GCCCTGCAGTACAACTCCAT | 85 |
|  | R | TGCCCTGCTGCGAGTAGGA |  |
| *KLF4* | F | CGCCGCTCCATTACCAAGAG | 82 |
|  | R | CACGATCGTCTTCCCCTCTT |  |
| *GAPDH* | F | GTGGACCTGACCTGCCGTCT | 153 |
|  | R | GGAGGAGTGGGTGTCGCTGT |  |
| **Primers for qRT-PCR** | | | |
| *Endo-OCT4* | F | GACAGGGGGAGGGGAGGAGCTAG | 142 |
|  | R | CTTCCCTCCAACCAGTTGCCCCAAA |  |
| *Endo-SOX2* | F | TGGCGAACCATCTCTGTGGT | 111 |
|  | R | CCAACGGTGTCAACCTGCAT |  |
| *Endo-KLF4* | F | ACAGTCTGTTATGCACTGTGGTTTCA | 84 |
|  | R | CATTTGTTCTGCTTAAGGCATACTTGG |  |
| *c-MYC* | F | CCAGCAGCGACTCTGAGGA | 75 |
|  | R | GAGCCTGCCTCTTTTCCACAG |  |
| *NANOG* | F | CAAGAAACAGAAGACCAGAACTGTG | 213 |
|  | R | CCATTGCTATTCTTCGGCCAGTTG |  |
| *GAPDH* | F | GTGGACCTGACCTGCCGTCT | 153 |
|  | R | GGAGGAGTGGGTGTCGCTGT |  |
